# Progress in Stem Cells-Based Replacement Therapy for Retinal Pigment Epithelium: In Vitro Differentiation to In Vivo Delivery

**DOI:** 10.1093/stcltm/szad039

**Published:** 2023-07-17

**Authors:** Santosh Gupta, Lyubomyr Lytvynchuk, Taras Ardan, Hana Studenovska, Ruchi Sharma, Georgina Faura, Lars Eide, Rama Shanker Verma, Ljubo Znaor, Slaven Erceg, Knut Stieger, Jan Motlik, Goran Petrovski, Kapil Bharti

**Affiliations:** Center for Eye Research and Innovative Diagnostics, Department of Ophthalmology, Institute for Clinical Medicine, Faculty of Medicine, University of Oslo, Oslo, Norway; Department of Ophthalmology, Justus Liebig University Giessen, University Hospital Giessen and Marburg GmbH, Giessen, Germany; Department of Ophthalmology, Karl Landsteiner Institute for Retinal Research and Imaging, Vienna, Austria; Laboratory of Cell Regeneration and Cell Plasticity, Institute of Animal Physiology and Genetics, Academy of Sciences of the Czech Republic, Libechov, Czech Republic; Department of Biomaterials and Bioanalogous Systems, Institute of Macromolecular Chemistry, Academy of Sciences of the Czech Republic, Prague, Czech Republic; Ocular and Stem Cell Translational Research, National Eye Institute, National Institutes of Health, Bethesda, MD, USA; Department of Medical Biochemistry, Institute of Clinical Medicine, University of Oslo, Oslo, Norway; Department of Medical Biochemistry, Institute of Clinical Medicine, University of Oslo, Oslo, Norway; Stem Cell and Molecular Biology, Laboratory, Department of Biotechnology, Bhupat and Jyoti Mehta School of Biosciences. Indian Institute of Technology Madras, Chennai, Tamil Nadu, India; Department of Ophthalmology, University of Split School of Medicine and University Hospital Centre, Split, Croatia; Research Center “Principe Felipe,” Stem Cell Therapies in Neurodegenerative Diseases Laboratory, Valencia, Spain; Department of Neuroregeneration, Institute of Experimental Medicine, Academy of Sciences of the Czech Republic, Prague, Czech Republic; Department of Ophthalmology, Justus Liebig University Giessen, University Hospital Giessen and Marburg GmbH, Giessen, Germany; Laboratory of Cell Regeneration and Cell Plasticity, Institute of Animal Physiology and Genetics, Academy of Sciences of the Czech Republic, Libechov, Czech Republic; Center for Eye Research and Innovative Diagnostics, Department of Ophthalmology, Institute for Clinical Medicine, Faculty of Medicine, University of Oslo, Oslo, Norway; Department of Ophthalmology, University of Split School of Medicine and University Hospital Centre, Split, Croatia; Department of Ophthalmology, Oslo University Hospital, Oslo, Norway; Ocular and Stem Cell Translational Research, National Eye Institute, National Institutes of Health, Bethesda, MD, USA

**Keywords:** retinal pigment epithelium, induced pluripotent stem cells, embryonic stem cells, cell delivery, differentiation

## Abstract

Retinal pigment epithelium (RPE) is a critical cell monolayer forming the blood-retina-barrier (BRB) and a permeable bridge between the choriocapillaris and the retina. RPE is also crucial in maintaining photoreceptor function and for completing the visual cycle. Loss of the RPE is associated with the development of degenerative diseases like age-related macular degeneration (AMD). To treat diseases like AMD, pluripotent stem cell-derived RPE (pRPE) has been recently explored extensively as a regenerative module. pRPE like other ectodermal tissues requires specific lineage differentiation and long-term in vitro culturing for maturation. Therefore, understanding the differentiation process of RPE could be useful for stem cell-based RPE derivation. Developing pRPE-based transplants and delivering them into the subretinal space is another aspect that has garnered interest in the last decade. In this review, we discuss the basic strategies currently employed for stem cell-based RPE derivation, their delivery, and recent clinical studies related to pRPE transplantation in patients. We have also discussed a few limitations with in vitro RPE culture and potential solutions to overcome such problems which can be helpful in developing functional RPE tissue.

## Graphical abstract

**Graphical Abstract:**
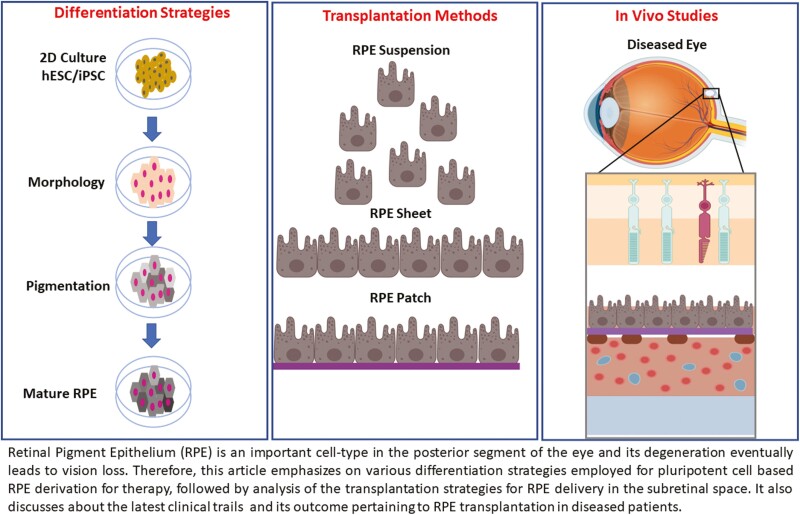

**Significance Statement**

This article provides concise information about the latest development in differentiation strategies used for deriving retinal pigment epithelium (iRPE) from pluripotent stem cells and various ways of transplanting iRPE. This article also describes limitations associated with in vitro culture of RPs and strategies to overcome such limitations. Finally, we have summarized all important clinical trials pertaining to the transplantation of iRPE in patients with macular degeneration and the future directions in the field.

## Introduction

Age-related macular degeneration (AMD) is one of the most prevalent forms of irreversible vision impairment in the aging population. Etiology of AMD is still not fully explained. However, several factors like age, genetic predisposition, immune system, and lifestyle have been associated with its development.^1,2^ Hallmarks of AMD pathological characteristic includes formation of drusen, deposit of lipids, and protein between the retinal pigment epithelium (RPE) layer and the choroid, which affects normal RPE physiological function and leads to photoreceptor degeneration. The late stage of AMD is broadly classified into 2 types, dry AMD and wet AMD.^3^ The dry form is associated with RPE loss while the wet form is characterized by abnormal blood vessel growth in the choroid, neo angiogenesis, followed by breaching of the blood-retina-barrier.^4^

The conventional therapeutic modality for the treatment of wet AMD is intra-vitreal anti-VEGF injection. Such a treatment approach is preventive rather than curative, as such in advanced disease stages RPE and photoreceptors still degenerate leading to vision loss and blindness. Recent advances in stem cell engineering have enabled the development of cell-therapy-based regenerative medicine for the treatment of AMD.^5^ Although these initial studies are highly significant, the need to develop a standard cell therapy with features that promote the transplanted tissue to integrate with the host tissue at the transplantation side is essential and requires multi-factorial regenerative, stem cell biology, and tissue engineering approaches.^6^ Recent studies suggest that injection of iPSC-derived RPE in the subretinal space leads to improper epithelization of the RPE monolayer. This could be owing to various reasons like loss of cell-to-cell contact leading to improper monolayer epithelization,^7,8^ Bruch’s membrane (BM) associated change in the functional behavior of transplanted RPE in the diseased environment,^9^ and de-differentiation of transplanted RPE cells leading to dysfunctional RPE. To overcome such undesired results, tissue engineering approaches are useful in the fabrication of patches that can be transplanted as an intact monolayer of iPSC RPE.^6^

To summarize, this article covers various aspects of stem cell-based derivation of RPE and its clinical advances. Initially, it highlights the in vitro differentiation of RPE using embryonic stem cells (ESC) and pluripotent stem cells (iPSC) employing a guided developmental strategy mimicking in utero RPE development. Further, we describe the differentiation strategies employed for RPE derivation including spontaneous, growth factors and small molecule-based approaches. Then, we discuss different methods of RPE transplantation and the limitations associated with different strategies. Finally, we discuss recent findings from the clinical trials of pRPE transplantation in patients suffering from AMD and other retinal diseases.

## In Vitro Differentiation of RPE

Several advancements have been made in the field of RPE derivation using iPSC and embryonic stem cells (ESC).^10,11^ While the use of ESC encompasses ethical concerns, iPSCs avoid such issues, and they present advantages like allogenicty and associated immune competency.^12,13^ Therefore, in recent years, interest in iPSC-based RPE derivation has been high and this approach has been explored recently in clinical trials where iPSC-derived RPE have been transplanted in AMD patients as a cell therapy module or via scaffold-based RPE patch.^14^ However, such studies have been made on a small scale and as a proof-of-concept. iPSC-based RPE derivation in itself has many challenges, which include the long process of differentiating iPSC to RPE, which can vary anywhere between 2 and 6 months.^6^

Recent clinical trials with stem cell-derived RPE includes ESC-derived RPE used for the treatment of geographical atrophy (the dry form of AMD),^15^ ESC-derived RPE patches in wet AMD patients,^16^ and an iPSC-derived RPE sheet in a patient with wet AMD.^17^ However, all the clinical trials conducted with ESC or iPSC-derived RPE relied on the spontaneous derivation of RPE during embryoid body culture and pigmented cells for further culture and expansion. This process usually takes between 4 and 6 months to achieve transplantable RPE. Therefore, several recent articles have focused on the differentiation aspect in order to reduce the differentiation time. Sharma et al. used small molecule inhibitors and growth factors to derive RPE and further fabricated a patch for transplantation in rat and pig models to study the efficiency of the RPE patch attachment and function of the transplanted construct.^18^ The authors in this study divided the differentiation strategy into 3 parts, ie, RPE induction, commitment, growth, and achieved the differentiation in 6-8 weeks. Similar strategies have been used in other publications where the stages of differentiation recapitulate the stages of RPE development in vivo.^18-22^ This becomes more relevant as the RPE commitment in vivo usually takes less time compared to the in vitro protocol being used to derive RPE.

In a recent study, to mimic in utero development of RPE, iPSC was differentiated into RPE using a protocol in 3 stages, including a series of small molecules and growth factors in 45 days.^23^ A common strategy is also the use of SMAD inhibition in the first stage to induce anterior neuroectoderm. It has been shown that during embryonic development RPE arises from the anterior neuroectoderm (ANE).^24^ Research by Surmacz et al. showed that the SMAD signaling pathway is inhibited during this process.^25^ Therefore, the use of SMAD inhibition to derive the precursor cells of RPE represents one approach to deriving RPE cells rapidly. From ANE, a specialized group of cells leads to eye field development eventually leading to the formation of eye structures having most of the cells of the posterior and anterior eye including RPE cells. During eye field development, a number of cell signaling pathways are either active or remain supressed.^24^ Oh et al. used BMP4 molecules, as BMP signaling pathway is activated during the process of eye field development. And finally, the RPE fate was further induced by treating cells with ACTIVIN A, a molecule shown to be essential in RPE induction.^26^

Recently another study published by Kuroda et al. extensively studied the role of FGF pathway inhibition in RPE derivation.^27^ They showed that direct inhibition of FGF and MEK signaling pathways was sufficient to induce RPE differentiation. They further showed that FGF and MEK inhibition was sufficient to overcome the need to inhibit WNT and Nodal signaling, which are essentially downregulated during the differentiation of RPE from RPE-specified progenitor cells.^27^

### ANE Formation

Results from previous publications have shown that inhibition of SMAD signaling using combined BMP inhibitor and TGF-β/ACTIVIN/NODAL inhibitors, referred to as dual SMAD inhibition can efficiently induce ANE formation.^25^ Using either BMP inhibitor^28^ or TGF-β/ ACTIVIN/NODAL inhibitor^29^ alone can induce neural differentiation. It has been shown that iPSC when cultured in FGF-free media exhibits spontaneous differentiation into RPE^30^ even quite efficiently when using a feeder-free system.^27^

### Eye Field Induction

Studies in zebrafish and chick embryos demonstrated that BMP signaling promotes forebrain development and inhibits eye field formation.^31^ In a study using iPSC, it was shown that inhibiting of BMP signaling pathway using a small molecule inhibitor promotes RPE differentiation.^27^ WNT/B-CATENIN signaling pathway was shown to play an active role in the RPE-specific gene expression at the late eye field stage.^32^ Furthermore, ectopic expression of WNT/B-CATENIN in optic vesicle resulted in the conversion of the whole optic vessel into RPE,^32^ suggesting an important role of the WNT/B-CATENIN signaling pathway in the eye field development and eventual formation of RPE.^32^

### RPE Specification

RPE specification involves the activation of several pathways, one of which involves ACTIVIN A signaling. A study using chick explants showed that in the absence of extraocular mesenchyme signaling, ACTIVIN A promotes RPE-specific gene expression and downregulation of neural-specific gene expression.^33^ In recent years, the use of ACTIVIN A has been extensively seen for the derivation of RPE using iPSC/ESC.^34^ This is owing to its role in the commitment and specification of progenitor cells towards RPE lineage.

### RPE Maturation and Growth

Once the RPE-specified cells are achieved with characteristic pigmentation, the cells need to be cultured for their maturation and growth. In various studies of RPE derivation, the last stage which is growth and differentiation, the major focus is to maintain the phenotype (morphology), function, and cells specific gene and protein expression.

## Differentiation Strategies for RPE

**Figure 1.**
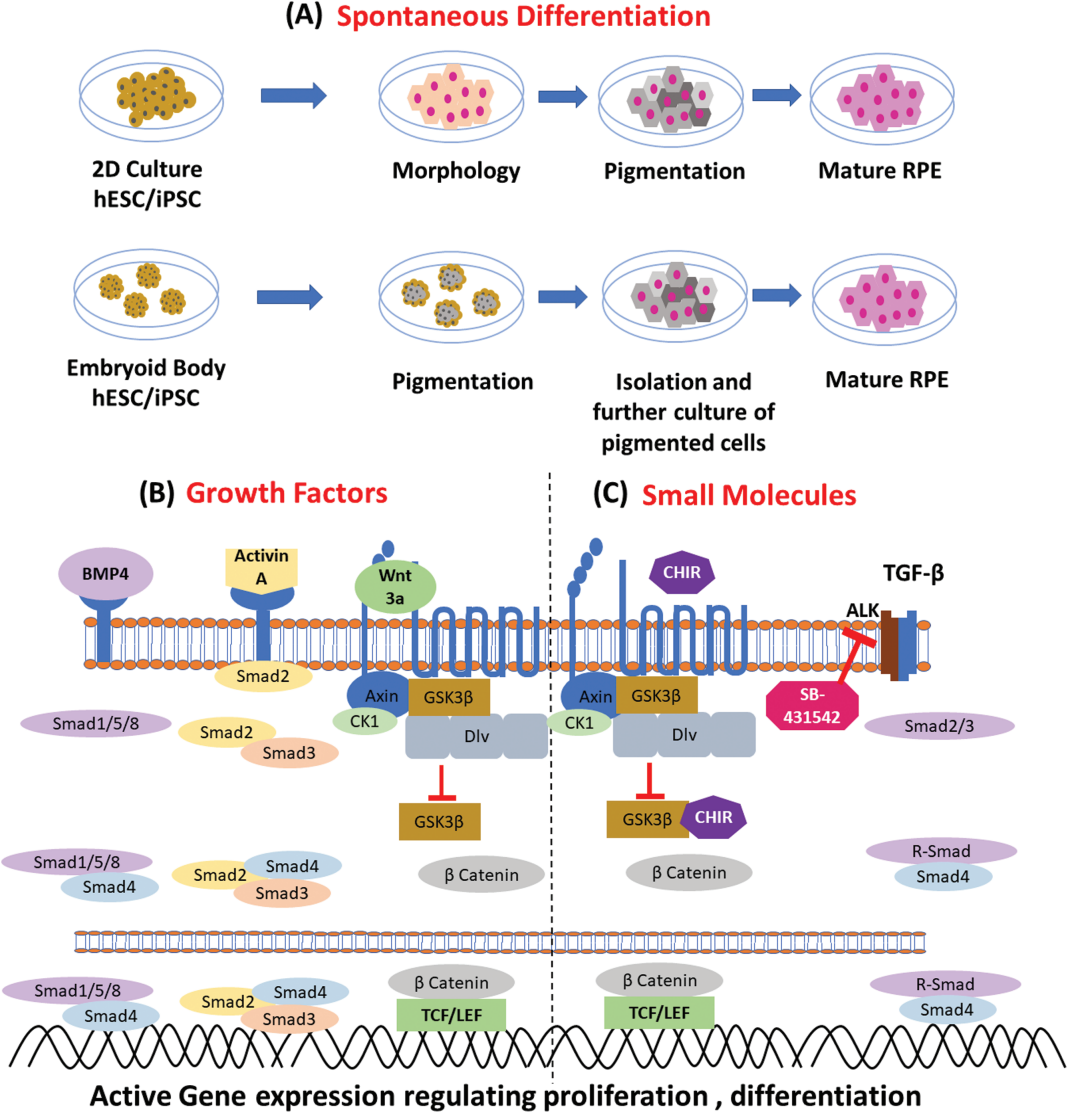
Schematic representation of differentiation strategies of RPE. (**A**) Spontaneous differentiation describing two common approaches i.e., 2D culture and embryoid body-based RPE derivation. (**B**) Growth factors-based differentiation which include representative growth factors like BMP4, ACTIVIN A, and WNT3a with its downstream cells signalling pathway and activation of signalling specific gene transcription. (**C**) Small molecules like CHIR 99021 and SB-431542 activating WNT signalling pathways and inhibiting TGFb1/ALK5 respectively.

Derivation of RPE from ESC and iPSC using various methods has been published (Table 1). These include the method of spontaneous differentiation of ESC or iPSC as adherent or suspension culture or using defined growth factors and small molecules mimicking in utero developmental process of the eye (Fig. 1). Spontaneous differentiation of stem cells into RPE is heterogenous and takes a long time (usually 4-6 months of in vitro culture) depending on the strategy being used for RPE derivation, that is, adherent (2D) or suspension (3D) where the first stage is initiated using embryoid bodies in suspension culture and subsequent stages in 2D. The use of defined media with growth factors and small molecules in combination further reduced the overall RPE derivation time to around 3.5 months.^18^ Such methods are controlled and less heterogenous compared to the spontaneous differentiation strategy and therefore require further refinement of the procedures to reduce the differentiation time and increase the purity and cellular homogeneity with functional maturation of the derived RPE.

**Table 1.** Tabular information on differentiation protocol for pluripotent stem cells (ESC, iPSC) based RPE derivation.

Cell type	Differentiation method	Differentiation factors	Differentiation time	Functionality	In vivo transplantation	Route	References
hESC	Spontaneous	β-mercaptoethanol, 13% serum replacement (SR)	6-9 months	CRALBP and BEST staining In vitro phagocytosis	N/A	N/A	^30^
iPSC	Spontaneous	20% Knock-out serum replacement, β-mercaptoethanol, fibroblast growth factor (zfbFGF).	18 weeks	PAX6, MITF immunostaining, in vitro phagocytosis Optokinetic	1 × 105 cells in SCID mice	Subretinally	^35^
hESC	Directed, growth factor	14% KO serum replacement, NIC, Activin A, TGF-β1, SB431542, bFGF,	6-10 weeks	MiTF-A and RPE65 immunostaining, immunostaining, in vitro phagocytosis	0.6-1 × 105 cells in RCS rats.	Subretinally	^34^
iPSC	Directed, growth factor	Nicotin-amide, Noggin, Dkk-1, IGF-1, bFGF, ActivinA, SU5402, CHIR99021	6-8 weeks	PAX6, MITF immunostaining, in vitro phagocytosis	N/A	N/A	^36^
iPSC	Directed, growth factor	5% KOSR, N2, insulin, ITS, b-ME, bFGF, noggin, SB, sonic hedgehog(shh)	6-8 weeks	PAX6, MITF, RPE65, ZO-1 immunostaining,	N/A	N/A	^22^
iPSC	Directed, growth factor	Noggin, Dkk1, IGF1, nicotinamide, 3-aminobenzamide, bFGF, Activin A, SU5402,	44 days	MITF and ZO-1 immunostaining, rod outer segment phagocytosis	N/A	N/A	^20^
iPSC	Directed, growth factor	5 % foetal bovine serum (FBS), N2, taurine, hydrocortisone, triiodothyronine, IGF1, Dkk1, Noggin, bFGF, B27,	90 days	MITF, OCC, BEST, and ZO-1 immunostaining, rod outer segment phagocytosis	N/A	N/A	^19^
iPSC	Directed, growth factor And small molecules	20% Knock Out serum replacement, b-mercaptoethanol, LDN-193189, SB-431542, BMP4, Activin A,	45	RALBP, ZO-1, and MERT immunostaining	N/A	N/A	^23^
iPSC	Directed, growth factor And small molecules	N2, B27, LDN-193189, SB431452, CKI-7 hydrochloride, and IGF-1, PD0325901, nicotinamide, Activin A, taurine, hydrocortisone, triiodo-thyronin,	77 days	ZO-1 immunostaining, CRALBP, and BEST1 expression, transepithelial resistance (TER), phagocytosis, VEGF secretion	Rat and—(100 000 cells) and the iRPE patch (10 000 cells) pig model—(100 000 cells) transplantation	Subretinally	^18^
iPSC	Directed, small molecules	CKI-7, SB-431542,	60days	MITF, ZO-1 immunostaining	N/A	N/A	^36^
iPSC	Directed, small molecules	Chetomin, nicotinamide, B27, KnockOut serum	35 days	RALBP, RPE65, BEST immunostaining, rod outer segment phagocytosis, VEGF secretion	5 × 104 cells NOD-SCID	Subretinally	^37^
iPSC	Directed, small molecules	IWR1e, CHIR99021 and SAG, fungizone, 20% KSR, 10% FBS, retinoic acid	60-100 days	CRALBP and BEST1 immunostaining, rod outer segment phagocytosis, PEGF secretion	1 × 10^5^ in RCS rats	Subretinally	^38^
iPSC	Directed, small molecules	PD0325901, PD173074, Y-27632, CKI-7	31 days	PAX6 immunostaining, PAX6 and MITF expression	N/A	N/A	^27^

### Spontaneous Differentiation

In 2004, Klimanskaya et al.^30^ reported the first study about the spontaneous development and isolation of RPE from hESC both in the adherent (all stages) and suspension (EB) culture (first stage followed by 2D culture of subsequent stages) method.^30^ Using adherent culture condition with or/without MEF, pigmented cells were observed after 6-8 weeks while using the EB-based method, pigmented cells were observed in 4-8 weeks. It should be noted that all cells under EB culture had pigmentation by 6-9 months. The limitation of this method is the heterogeneity and the low efficiency of RPE derivation. However, the derived RPE was transcriptionally similar to freshly isolated fetal RPE as compared to an adult human RPE cell line (ARPE19).

In 2009, Carr et al. confirmed the ability of iPSC to differentiate into functional RPE.^35^ The authors showed the protective effect of iPSC-derived RPE preventing and slowing down photoreceptor degeneration in a retinal dystrophic rat model. In this study, iPSC differentiation was done in adherent cultures, RPE progenitors were observed 4 weeks from the day of differentiation induction and were further cultured for another 14 weeks for RPE maturation and expansion.

The molecular mechanism causing the spontaneous development of RPE from ESC or iPSC is not clear. However, in most strategies, following the removal of basic FGF, marks the induction of differentiation of ESC and iPSC toward pigmented phenotype. During in utero development of the eye field, a committed population of cells, from which RPE originates, also enables inhibition of bFGF signals. This results in the lineage commitment toward pigmented epithelial cells along with an intricate play of other cells signaling molecules and pathways.

### Growth Factors

One of the earliest reports of RPE derivation using ESC involved the culture of ESC on a stromal cell line (PA6) as inducer.^39^ A similar study was performed using primate ESC with stromal cell layer support to derive RPE in 2003.^40^ It was unclear which molecules secreted by stromal cells were playing a role in the induction of RPE differentiation. However, these studies emphasize the importance of some secreted molecules and their role in the induction of differentiation. However, the published protocols are heterogenous and non-reproducible with low derivation efficiency. Over time, more protocols with controlled growth factors and small molecule combinations have been developed which mimic the in utero development of the eye and at the same time focus on reducing the differentiation time from stem cells to RPE cells. The first study utilizing the WNT signaling pathways in RPE development mimicking in utero was published by Aoki et al. in 2006.^41^ They used WNT2B recombinant protein to show that the efficiency of RPE differentiation was increased when cultured on the stromal cell line.

In a different study, a direct ESC-based differentiation approach for RPE derivation was used employing nicotinamide and ACTIVIN A.^34^ Although the role of nicotinamide is not yet clear in RPE differentiation, it has been used extensively in most of the differentiation protocols. ACTIVIN A is a member of the TGF β superfamily, acting primarily through SMAD2/3 proteins, and regulates a variety of cellular features and functions such as cell proliferation, differentiation, apoptosis, metabolism, and repair response.^42^ ACTIVIN A also presumably plays an important role in the RPE development during embryogenesis.^33,43^

Several protocols were developed using the serum-free embryoid body-based differentiation method. Embryoid body-based approach favors neuronal precursors formation and subsequent derivation of RPE. Proteins like Dkk1 (Wnt/β-catenin signaling pathway inhibitor)^44^ and Lefty A (antagonist of nodal signaling pathways)^45^ were used for enhancing neuronal precursor for RPE differentiation from ectoderm-derived neural progenitors. Dkk1 has been used in combination with other factors to induce RPE from hiPSC.^46^

Gradually, several protocols have been published that utilize the sequential addition of growth factors and small molecules to induce various stages of RPE development as observed during in utero developmental processes. In a study by Zahabi et al.,^22^ Noggin, SB43154, and retinoic acid were added to induce neuronal differentiation followed by the addition of FGF and sonic hedgehog (Shh) factor over a period of 40 days of differentiation.^22^ Further, maintenance and maturation over 50 days yielded more than 50% of the RPE population. Similarly, Buchholz et al. developed a protocol using previously validated neuronal retinal progenitors-inducing factors like Noggin, IGF1, Dkk1, and bFGF followed by a combination of known RPE-inducing factors nicotinamide, activin A, vasoactive intestinal peptide, and SU540.^20^ This protocol yielded over 80% of RPE cells using ESC and 63% using iPSC.

Defined media with serum-free conditions were shown to produce high purity of RPE from iPSC.^47^ Serum-free media are clinically more attractive as they overcome the ­xenogeneic nature of the serum used along with possible cross-contamination of zoonotic microorganisms. Most importantly, they overcome the heterogeneity in serum parameters which differs from batch to batch. Therefore, for clinical transplantation protocols, defined media conditions for the reproducible derivation of RPE are necessary.

In a more recent study, IGF1, Dkk1, Noggin, bFGF, B27, and N2 were used in the defined composition. Pigmented cells started to appear after 20 days and 90% of RPE cells were present after 90 days of differentiation based on positive staining for PMEL17.^19^ In another study, a combination of small molecule inhibitor and recombinant proteins was used for the directed differentiation of iPSC into RPE in 45 days with more than 90% of CRALBP positive RPE cells. The authors used a combination of the small molecules LDN-193189 and SB-431542 (SMAD inhibitors) to induce ANE. Upon ANE induction, BMP4 and ACTIVIN A were used to induce differentiation towards RPE.^23^ Such a dual use of small molecule inhibitors and recombinant protein factors is useful in reducing the overall derivation time of the differentiation protocol.

The development of clinical grade RPE is essential for use in human clinical trials and therefore requires differentiation protocols that have regulatory approval for the chemical and biological factors used for inducing differentiation. In this regard, the study by Sharma et al. developed a protocol that took 112 days for the derivation of clinical grade fully polarized functional RPE under defined conditions commonly used in the laboratory and even in clinical practice for biological product development.^18^ To induce RPE cells, neuro ectodermal cells were treated with a combination of N2, B27, LDN-193189, SB431452, CKI-7 hydrochloride, IGF-1, and PD0325901 for 3 weeks followed by maintenance in a condition media with N2, B27, nicotinamide and ACTIVIN A for 3 weeks and finally transfer into growth media containing taurine, hydrocortisone and triiodo-thyronin. This process reduced the overall differentiation time to 77 days for the derivation of clinical grade RPE and RPE patches for application in clinical studies. The controlled media composition with a mixture of small molecules and recombinant proteins resulted in RPE with 96% of cells expressing the RPE progenitor gene PAX6.^18^

### Small Molecules

Small molecules-based differentiation procedures offer advantages over growth factor-based protocols owing to their non-biological origin, stable activity, based protocol lots, and cost efficiency.^48^ With respect to RPE derivation from stem cells (ESC and iPSC), exclusive small molecules-based procedures are limited compared to conventional growth factor and small molecule combination-based protocols.

Osakada et al. used a combination of casein kinase I inhibitor CKI-7, the ALK4 inhibitor SB-431542 to study the RPE differentiation capacity in serum-free embryoid-like aggregate method.^36^ They observed pigmented cells on day 40 and by day 60 the cells displayed hexagonal morphology with phagocytic ability. Although, the protocol was small molecule-based, the derivation efficiency was low (around 18%). The cells were functional and displayed molecular expression of RPE-specific proteins like RPE65, ZO-1, and CRALBP.

In 2015, Maruotti et al. described an exclusive small molecules-based approach in the derivation of RPE from iPSC by a direct differentiation approach.^37^ Using a high-throughput small molecule library screen, they identified chetomin along with nicotinamide-producing PMEL17 positive RPE cells with an efficiency ranging from 46% to 60% at day 35. This small molecule-based approach resulted in induced RPE exhibiting morphological, molecular, and functional characteristics similar to native RPE.

Some reports used a small molecule-based approaches to derive RPE from iPSC and ESC, their differentiation protocol involved fetal bovine serum along with small molecules to induce differentiation. Although these approaches promote cell growth and proliferation, the use of serum derived from xenogeneic sources may exhibit qualitative and quantitative heterogeneity of growth factors from batch to batch. Therefore, a serum-free or growth factor-reduced knockout serum-based protocol involving small molecules is required for clinical application.

For example, a small molecule-based approach was explored to differentiate RPE from 3-dimensional embryoid body derived from hESC.^38^ They used IWR1e, CHIR99021, SAG, and fungizone. From day 12 onwards during the differentiation process, 10% FBS was used till pigmented cells were observed. In this protocol, the 2D or adherent-based approach yielded pigmented cells in 60 days whereas 3D-based approach did not show pigmented cells until day 100. By day 100, both 3D and 2D-based approaches yielded pigmented cells. However, the characteristic hexagonal morphology was observed at day 20 in both the 3D and 2D-based approaches. The authors claimed that subretinal transplantation of the ESC-derived RPE was safe and efficient to rescue retinal degeneration in RSC rats.

Recently in another study, an exclusive small molecule-based— activators and inhibitors of cell signaling pathways known to be actively associated during RPE development in utero were used.^27^ They showed that by inhibiting MAPK pathways using inhibitor PD0325901 and FGF signaling by inhibitor PD173074, pigmented cells appeared by day 14-21 which were further saturated by day 31. The differentiation efficiency was greater than 98% as assessed by PAX6 and MITF markers. The authors further showed that by inhibiting FGF/MAPK signal inhibition, a requirement of Wnt and Nodal signal inhibition was eliminated. Thus, providing a small molecule-based inhibitory approach in the derivation of RPE in a serum and feeder-free system which has potential clinical translation relevance. However, in this study, the authors did not perform functional studies of derived RPE cells or in vivo transplantation studies to show whether such cells are actively able to perform critical functions like phagocytoses, exhibit epithelial resistance, and most importantly, the therapeutic potential of such RPE in a preclinical model upon transplantation.

## Delivery of RPE to the Subretinal Space

The first study of RPE transplantation in an animal model was performed by Li and Turner in 1988 when they transplanted RPE in the Royal College Surgeon (RCS) rat model of retinal dystrophy as a proof-of-concept study showing the utility and biological significance of the transplantation of RPE in the potential treatment of blindness.^49^ The rationale behind the experiment was the aim to restore the function of degenerated retina, as it had been proposed by Dowling and Sidmen. In 1962, these 2 authors found that in RCS rats phagocytosis of photoreceptor outer segments (POS) by the RPE was dysregulated leading to the degeneration of the retina.^50^ Furthermore, D’Cruz et al., found that in RCS rats the *merTK* gene was mutated and this loss of function resulted in reduced capability of RPE cells for phagocytosis of POS followed by degeneration and death of photoreceptors.^51^ Based on these observations, Li and Turner performed an experimental study where RPE, obtained from black-eyed Long Evans rats, was transplanted in the subretinal space of RCS rats to study potential changes in retinal degeneration. However, it should be noted that prior to the work of Li and Turner, few other work validating the feasibility of RPE transplantation in rabbit and primates model^52^ was performed, thus suggesting the clinical suitability of such procedure for the treatment of retinal/RPE-related disorders.

**Figure 2.**
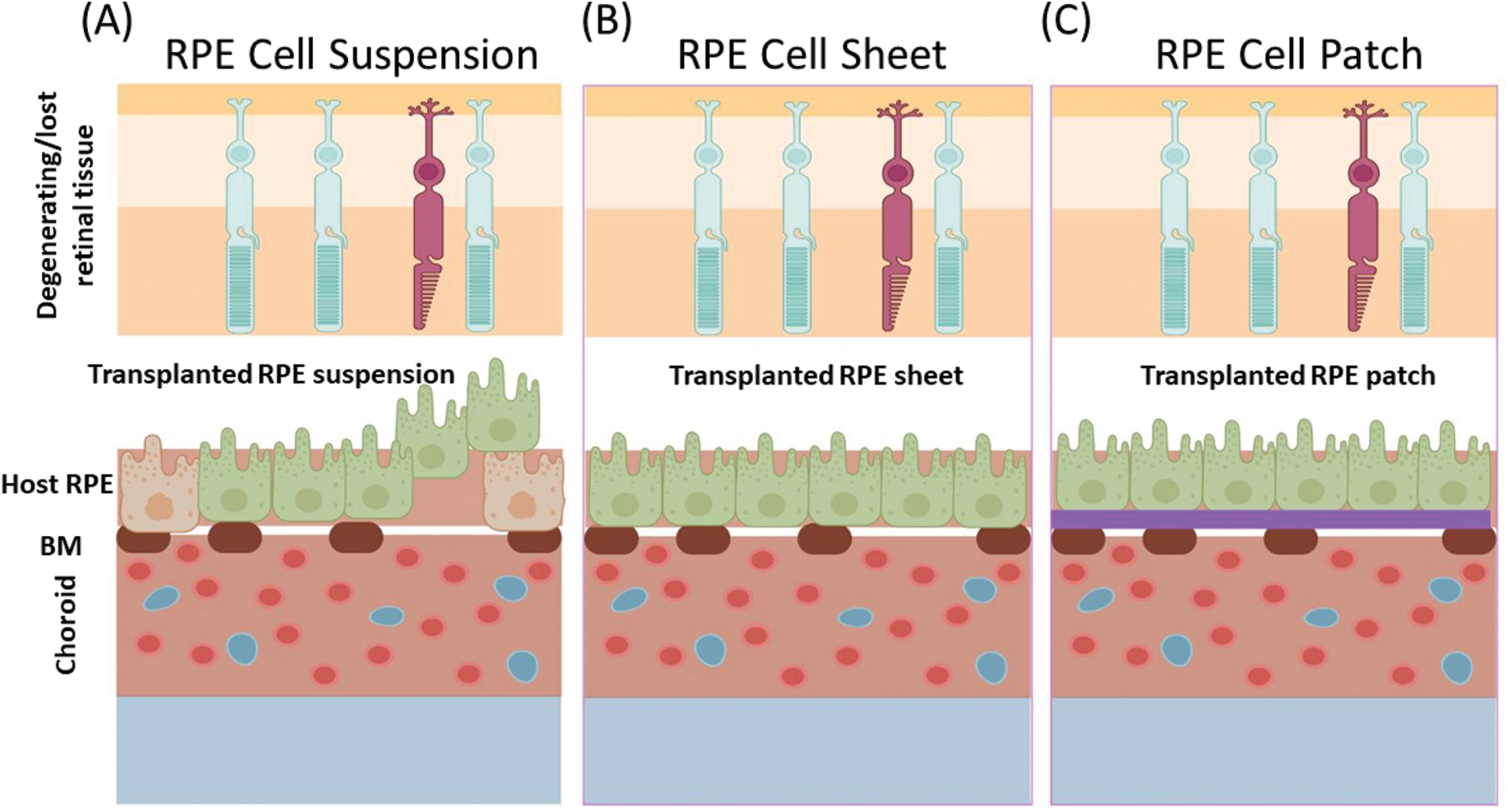
Schematic representation of the RPE delivery strategies explored so far. (**A**) RPE cell suspension-based delivery where cells are injected in the subretinal space using a specialized blunt syringe. (**B**) RPE cells sheet-based delivery, where RPE cell sheet is fabricated and delivered to the implant site using a specialized delivery vehicle. (figure 1) RPE cell patch-based delivery, where the RPE cells are cultured on a biocompatible polymer-based film or nanofibrous scaffold and followed by the subretinal delivery at the site of implantation using a specialized delivery device. BM, Bruch Membrane; RPE, Retinal Pigment Epithelium.

Before RPE transplantation, surgical technique-based approach to remove diseased RPE was being explored for the treatment of neovascularized AMD.^53^ Consequently, several studies have since been undertaken to study the therapeutic efficacy of transplanted RPE in various types of blindness-related disorders. Basically, 3 strategies have been explored so far with respect to RPE transplantation. (1) RPE cell suspension transplantation; (2) RPE sheet transplantation; and (3) RPE transplantation using support substrates (Fig. 2).

### RPE Cell Suspension

Transplantation of RPE cell suspension through small retinotomies allows comfortable delivery of limited cell amount into the subretinal space. Subretinal transplantation of a human RPE stem cell-derived RPE (hRPESC-RPE) suspension was found to preserve vision in an RSC rat model of RPE cell dysfunction.^54^ In preclinical animal model of chemically induced RPE loss, polarized cell monolayer was found after transplantation of hESC-derived RPE cell suspension in subretinal space with RPE-free Bruch’s membrane.^55^ The first study of human ESC cell line (MA09-hRPE) derived RPE suspension transplantation in human patients of AMD showed improvement of visual acuity in a few patients indicating that the transplanted RPE cells improved function of the adjacent photoreceptor cells.^56^ Although, there was concern about the safety and oncogenicity related to the use of ESC cell lines for deriving RPE,^57^ over the period of time, various studies related to ESC-derived RPE and long-term safety reports published periodically have substantiated the safety and therapeutic effect of the ESC derived RPE cells for the treatment of macular degeneration.^58-60^

Owing to ethical reasons, iPSCs have been recently used for the derivation of RPE for clinical applications in the future. A recent study reported safety and efficacy of clinical-grade human iPSC-derived RPE in a preclinical animal model. In this study, the iPSC-RPE cell suspension survived upon subretinal transplantation for 19 weeks and maintained the visual function of the adjacent photoreceptors for 15 weeks. While the use of iPSC for RPE derivation offers advantages compared to ESC.^61^ There is still a certain limitation in the clinical application that should be overcome. It was shown that the allogenic iPSC-derived RPE failed to graft upon subretinal transplantation in rhesus macaques. The authors showed that there was an early T-cell response followed by B-cell response at the site of RPE implantation. The host RPE and choroid were found disrupted in the immediate vicinity of the RPE suspension graft with fibrotic features in the subretinal space.^62^ A practical solution to the graft rejection due to immunological response of the transplanted iPSC-derived RPE was shown recently, where the authors proved that MHC mismatching caused the immune attack on the iPSC-RPE.^63^ By developing MHC-matched allograft iPSC derived RPE, the authors showed that T cells failed to elicit response to the MHC-matched homozygous iPSC-derived RPE. Another study related to the tumorigenicity of the iPSC-derived RPE were conducted in a mouse model to assess their safety.^64^ It was found that the subretinal injection of 1 × 10^6^ iPSC-derived RPE cells had no observable tumorigenic effect up to 15 months in a nude mouse model. Furthermore, 0.8-1.5 × 10^4^ iPSC-derived RPE cells in a collagen film also did not exhibit any tumorigenic phenotype up to 12 months. These observations indicate the safety use of iPSC-derived RPE for potential clinical applications.

Despite the latest advancements, there are still certain limitations with the induced RPE cell suspension delivery method. Injection of the RPE cell suspension is surgically faster and less invasive than the other techniques. However, several disadvantages were revealed, such as regress of the suspension into the vitreous cavity, limited adherence of RPE cells to the aged Bruch’s membrane, improper coverage of the damaged subretinal space, incorrect apicobasal orientation, and failure to form a confluent monolayer.^65^

### RPE Cell Sheets

RPE as a graft performs physiologically better and survives when transplanted as a sheet than as a suspension.^66,67^ There are various factors that seems to affect this observation including the environmental factors, especially in the case of diseased patients, where the basement membrane is compromised along with the change in the extracellular matrix.^68^ The main advantage of a self-supporting RPE cell sheets and also an RPE cell monolayer supported by an artificial substrate is that the RPE cells are implanted in a highly differentiated state.^66^ The grafted cells in ESC-RPE sheet were found to survive without phenotypic alterations in the eye of non-human primates and rodents.^69^

RPE cell sheets also provide the native extracellular matrix, which may prevent cells from undergoing apoptosis occurring in the case of cellular suspension transplantation.^70^ The formation of a tight barrier is a characteristic of RPE in vivo, which can be achieved when using RPE cell sheet than cell suspension transplantation.^71^ In this regard, Kamao et al. developed a human iPSC-derived RPE sheet displaying typical RPE markers, tight junction, exhibiting polarized secretion of growth factors, and showed phagocytic ability and gene expression pattern similar to native RPE. iPSC-RPE sheet upon transplantation in non-human primates showed no rejection and no tumor formation, thus proving the safety of the RPE sheet for clinical applications.^72^ Another study by Liu et al., showed that (hRPESC-RPE) sheet upon transplantation in non-human primates under immunosuppression did not undergo epithelial to mesenchymal transition (EMT).^73^ EMT is one of the features of RPE in diseased conditions leading to its detachment and loss. RPE identity of transplanted RPE graft was maintained, and RPE sheet transplantation was thus preferred to RPE cell suspension delivery. A review by Zhou et al. showed that the presence of EMT in RPE cells is a significant predictor for disease prognosis.^74^ Recently, a clinical trial study of 1 patient was published, where iPSC derived RPE sheet was transplanted in an AMD patient with wet AMD. Although the best-corrected visual acuity did not improve or worsened along with the development of cystoid macular edema, the RPE sheet remained intact for a period of 1 year.^75^ Cell sheet technology, although interesting and having certain positive aspects, may come with few disadvantages, such as curling of the RPE cell sheet from the manipulation point of view, the fragility of the cell sheet upon transplantation, perforation or breakage of the cell sheet leading to failure of the improper grafting, physiological function, and eventually failure of the transplanted graft.

### RPE Cell Patch

Another delivery technique includes RPE cells grafted on a tissue-engineered scaffold. Such a substrate should mimic the properties of Bruch’s membrane,^76^ and thus enables the renewal of the functioning epithelium by the monolayer organization and correct polarization of the RPE Substrate-based technology for RPE cultivation has already been known for several years. Many synthetic and biological substrates have been applied for subretinal transplantation of the RPE patch.^77^ However, only few of them met at the same time parameters for successful transplantation and renewal of the RPE such as porosity ensuring sufficient permeability for fluids, low thickness comparable, or less than the thickness of Bruch’s membrane, degradability, and feasibility for surgical manipulation.^78-81^

Therefore, a suitable method of preparation of RPE monolayer sheet based on a thin supporting biomaterial layer may overcome some of the limitations of the cell sheet technology with respect to RPE transplantation and at the same time provide appropriate microenvironment for the attachment, metabolic and physiological functioning of the RPE monolayer on the patch. Recently several studies related to the development of a scaffold-based RPE patch have been published.

Stanzel et al. showed that cells survive and maintain their apicobasal polarity in the rabbit retina when cultivated on polyester substrates.^82^ A clinical trial data was published, in which a polyethylene terephthalate (PET) membrane was used for the fabrication of hESC-RPE patch. This patch was transplanted in 2 patients with exudative AMD. A 12-month follow-up showed that both the patients had visual acuity gain of 29 and 21 letters, respectively, which indicates successful integration and proper maintenance of the adjacent photoreceptor cells.^16^ PET membrane supported the survival of hESC-RPE sheet in vivo but failed to preserve the photoreceptors and the inner retinal layers. It was found that the low permeability of applied scaffolds is the main factor of failure. Similar findings were observed by Fernandes et al. when using the ultrathin parylen C substrate seeded with hESC-RPE.^83^

The synthetic parylene substrate for fabrication of hESC-RPE patches was also employed to characterize the therapeutic efficiency in patients suffering from AMD. All four patients with the implant showed evidence of hESC-RPE integration with the host photoreceptors. There was no progression in the vision loss observed, 1 eye improved by 17 letters and 2 eyes demonstrated improved fixation.^84^ Another example of application of nanoengineered ultrathin parylene C scaffold is the study of a long-term effect of human iPSC-RPE patch as a polarized monolayer transplanted in an immunodeficient RSC rats. At an early time point, transplants remained as a monolayer, expressed RPE-specific markers, performed phagocytic function, and contributed to vision preservation. However, after 11-month RPE survival was observed in only 50% of the eyes. Loss of RPE monolayer characteristics was connected with peri-membrane fibrosis, immune reaction through the activation of macrophages (CD 68 expression), and the EMT transition of cells.^85^

Fibrous scaffolds prepared by electrospinning in comparison to track-etched polymer membranes offer three-dimensional surroundings, providing a continuous flow of nutrients during the cultivation of RPE cells.^86^ Hayes et al., reported that the architecture of the basal infoldings can influence transepithelial transport and RPE cell adhesion.^87^ Later on it was proved that native basal infoldings of human iPSC-derived RPE cells were found only on the porous nanofibrous membrane and not on track-etched membrane. However, other characteristics such as morphology, electrical properties, and expression of RPE markers were found similar.^18^

**Figure 3.**
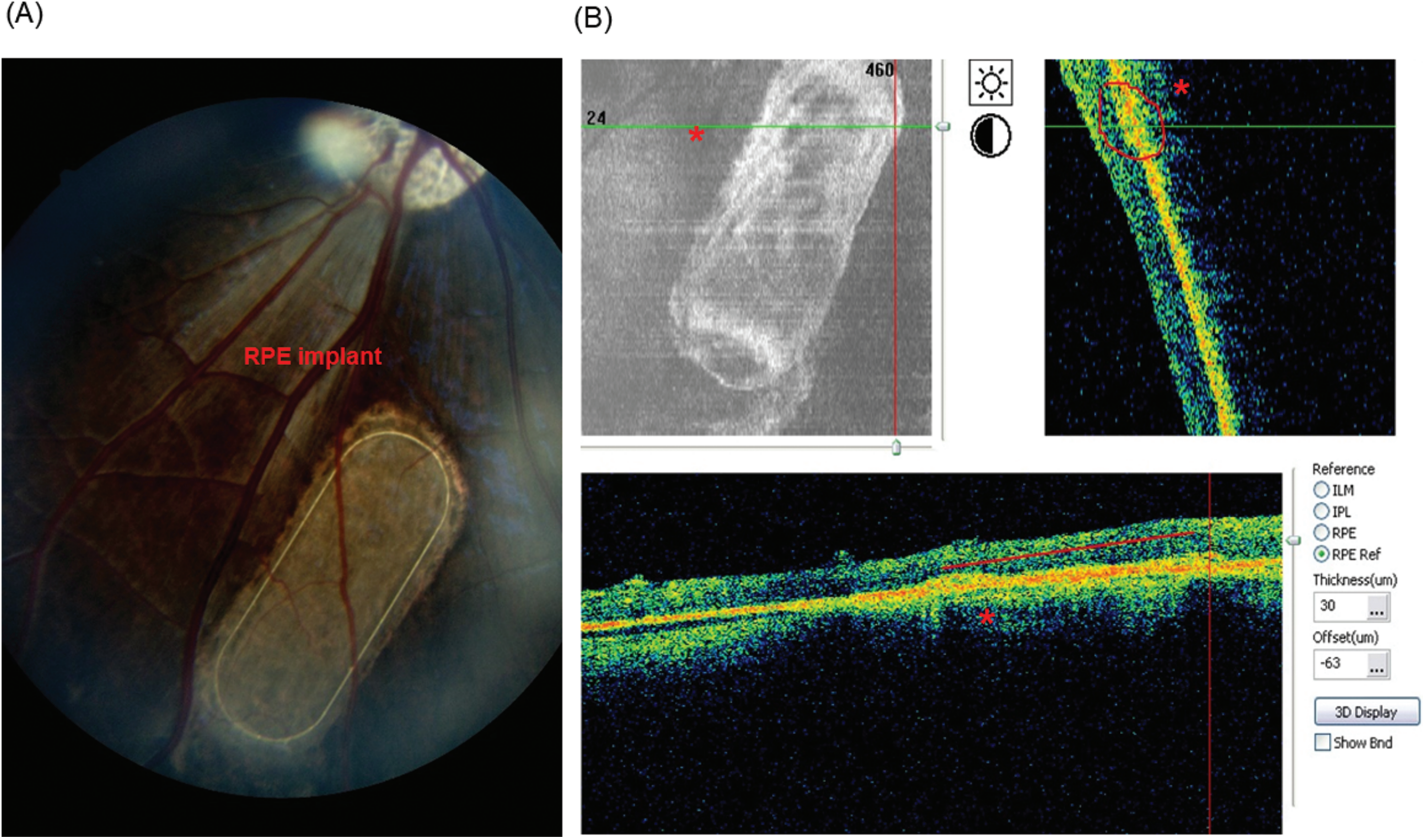
Non-invasive postoperative examinations of RPE implant by OCT (optical coherent tomography) and fundus photography (unpublished data from our laboratory). (**A**) Oval nanofibrous implant seeded with cultured porcine primary RPE cells (red arrows) and transplanted into subretinal space of minipigs eye was examined by fundus camera at 8 weeks after implantation and (**B**) by OCT at 4 weeks after implantation. ‘*’, Sub-retinally transplanted RPE implant in the minipig eyes.

Ultrathin nanofibrous scaffolds based on degradable polylactide (PDLLA) facilitated the physiological flow of nutrients through porosity over 70% and thus preserved the functioning of RPE cells.^88^ Ultrathin PDLLA nanofibrous membrane with thickness of 3-4 µm corresponds with thickness of Bruch’s membrane of 2-6 µm, however, the wet ultrathin membrane is difficult to handle during biological and surgical manipulations. Popelka et al. reported embedding of the peripheral frame into the nanofibrous PDLLA membrane that enabled the ultrathin membrane to be handled without irreversible folding and allowed the membrane to regain its flat shape when transplanted subretinally.^89^ This concept was successfully applied later in subretinal implantation of porcine RPE cultured on nanofibrous carrier (Fig. 3) and on human RPE cultured on enhanced nanofibrous membranes in minipigs.^90^ In another study, clinical grade iPSC-derived RPE was used for the first time to fabricate an iPSC-RPE patch using poly (lactic-co-glycolic acid) synthetic polymer-based nanofiber scaffold. The iPSC-RPE patch was tested in rodents and pigs for its safety and efficacy. RPE patch exhibited better integration and functionality in rats and in a porcine laser-induced RPE injury model mimicking AMD-like conditions.^18^ These studies of safety, functionality, and efficacy on rats, pigs, and humans underlines the utility of RPE patch and the rationale upon which the RPE patch has been fabricated.

Still, there are a few factors that require extensive studies related to the RPE patch-based transplantation method as degradability and the effect of patch adherence on the implanted site. In the aforementioned studies, the materials used for fabricating patch were synthetic polymers and they do not expose cell adhesion moiety. Therefore, study of the long-term RPE-patch interactions is essential, especially if the patch materials take from months to years to degrade completely.

It is therefore essential to consider the advantages and disadvantages of biodegradable vs. non-biodegradable scaffolds for RPE patch transplantation studies in vivo.^91^ In this regard natural biomaterials^92^ like collagen,^93^ gelatine, and synthetic polymers like PCL,^94^ PLGA^18^ which are biodegradable and non-biodegradable scaffolds like parylene,^66^ polytetrafluoroethylene^95^ have been used in RPE patch development. Biodegradable scaffolds are ideal due to their degradation within a biological environment in a controlled manner whereas non-biodegradable scaffolds ideally could persist in the body for years without undergoing degradation.^96^ This would not be desirable as tissues continually exist in an equilibrium of extracellular matrix turnover. Degradation of the artificial scaffold should match the rate of synthesis of new natural extracellular matrix by the cells. The dgradation product of the biodegradable scaffold is another aspect that must be considered. Synthesis reproducibility of the biomaterials (degradable and non-degradable) is also important for aching the desired manufacturing features and regulatory approvals. However, factors like mechanical properties of the scaffolds derived from natural polymers are often low and require further improvement by chemical and physical crosslinking mechanism or blending with other natural or synthetic polymers. On the other hand, biodegradable scaffolds derived from synthetic polymers offer better mechanical properties which can be fine-tuned depending on the cell and tissue type. The degradation profile of the synthetic biomaterials-based scaffold can be fine-tuned as a function of physical properties like its molecular weight.^96^ Nonbiodegradable scaffolds on the hand do not possess cell-binding properties, and are physically more hydrophobic, and biologically inert in terms of cell interaction. Therefore, the functional integrity of such scaffold might not produce desirable biological effects.

## Limitations of In Vitro Expansion of RPE

**Figure 4.**
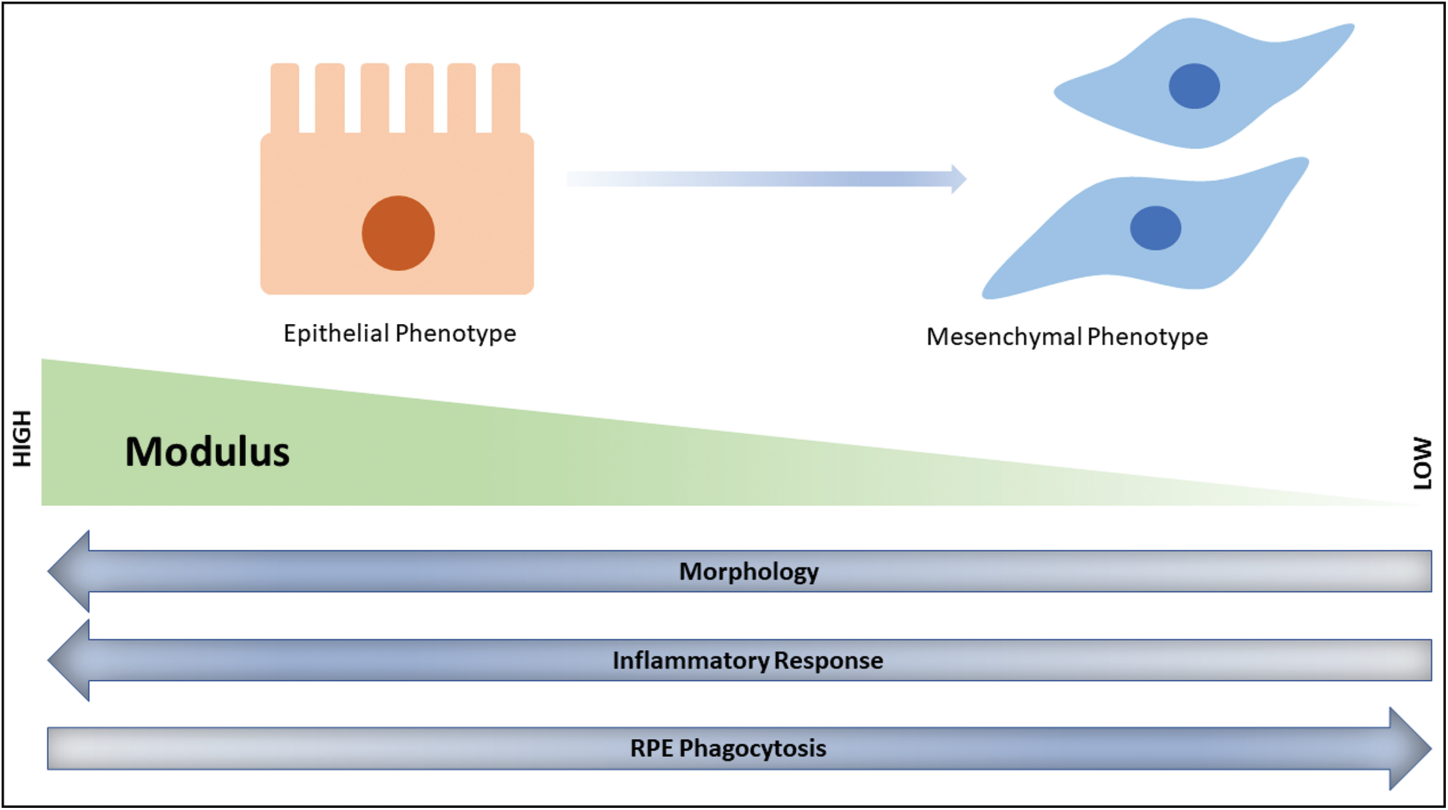
Schematic representation of the modulation of the functional behaviour of RPE when cultured in vitro. Loss of epithelial phenotype is observed which concomitantly associates with enhanced activity of TGF b cell signalling pathways through SMAD activation. In vitro expansion of biomaterials with high to low modulus affects various cellular phenotype and behaviour. Cells exhibits a higher inflammatory response when cultured on biomaterials with high elastic moduli. Similarly, RPE phagocytosis ability is efficient when cultured in low elastic modulus.

Another factor that influences the success of stem cell-derived terminally differentiated cells is the maintenance of the derived cells in their functional state. One of the features observed with almost all primary and stem cell-derived terminally differentiated cells is the induction of the de-differentiation process. These processes result in the change of the functional state of the cells and various factors have been associated with the induction of the de-differentiation process. These processes include EMT, induction of apoptosis, loss of functionality due to the absence of a suitable extracellular environment, and stiffness of the materials on which cells are cultured. Out of these, EMT transition has been extensively observed in cells including for human primary (hp)RPE. Such a process leads to the loss of functional properties of RPE cells, thus rendering them ineffective for transplantation. There are limited studies that focus on providing conditions like antioxidants or culturing RPE on a substrate with defined stiffness to prevent induction of de-differentiation processes. Controlling the de-differentiation process in terminally differentiated pRPE and hpRPE thus becomes essential in the preparation of human treatment trials use for AMD^97-99^ (Fig. 4).

### EMT Inhibition in Cultured RPE Cells

Loss of RPE differentiation appears to be critical for the induction of numerous retinal diseases. RPE dysfunction is connected with the presence of EMT when cells transform into mesenchymal phenotype.^100^ Potential mechanisms include impaired tight junctions, accumulation of misfolded proteins, and dysregulation of several signaling pathways and molecules. This is not desirable from the clinical application point of view. To control the de-differentiation process related to EMT, it is essential to understand various cell signaling pathways involved in the EMT induction and transition and possibly offering a solution by inhibiting the activation of certain cell signaling pathways which are overactive and result in EMT transition.^74^ Some of the pathways involving EMT are the TGF-β1 signaling pathway, NF-κB signaling pathway, Wnt signaling pathway, Akt signaling pathway, PPARγ signaling pathway, Notch signaling pathway, RAS signaling pathway.^101^ Although the role of the aforementioned pathways is cell dependent and is generally observed in cancer stem cells, the fundamental basis can be applied to inhibit the EMT transition in primary cells and iPSC-derived RPE. One of the transcription factors that have been shown to directly influence the de-differentiation process in RPE is SMAD, a downstream signaling molecule in TGF signaling pathways.^102^

### Substrate Stiffness for RPE Culture

The scaffold on which cells are cultured has specific young’s modulus and such mechanical properties influences the phenotype as well as function of the cells like adhesion, migration, cell-specific gene expression, loss of epithelial state, and even loss of stemness.^103,104^ To deliver RPE to the specific site and ensure transplantation and attachment of RPE, RPE-based patches are being explored and even clinical trials with ESC^6^ and iPSC^18^-derived RPE patch have been transplanted in rat, pig models, and human as a part of clinical trials for the safety studies. However, even in such studies, the stiffness of the scaffold or substrate is largely ignored. In a study where poly (ethylene glycol) diacrylate (PEGDA) scaffold of varying molecular weights from 3.4 to 20 kDa was fabricated with Young’s modulus varying from 60 kPa (low) to 1200 kPa (high) to understand the effect of stiffness on the de-differentiation and immune response of ESC derived RPE. It was found that RPE cultured on high stiffness had 3-fold more expression of IL6 and 2-fold higher expression of MCP-1 compared to low stiffness. These chemo-attractants attract microglial cells, immune cells of the retina, and might promote inflammatory response in vivo. Similarly, RPE displayed a more homogenous morphology when cultured on high Young’s modulus compared to low Young’s modulus.^105^ Similarly in another study, the effect of substrate elastic modulus was studied on the ability of RPE to phagocytosis. As in the case of AMD, the Bruch’s membrane, on which RPE adheres, becomes stiffer with age and therefore may affect the function of RPE like phagocytosis.^106^ In this study, ARPE19 was cultured on laminin-coated polyacrylamide substrates of varying elastic modulus for 14 days. It was found that phagocytosis decreased when cells were cultured on a stiffer substrate compared to a soft substrate.^107^ Thus, showing the importance of scaffold design focusing on the mechanical property as it influences functions like phagocytosis, morphology, and even promotes or prevents dedifferentiation.

## Clinical Trials for AMD Treatment Employing RPE

Transplantation of pluripotent stem cell-derived (ESC or iPSC) RPE is the first type of cell transplantation performed on the human for the treatment of AMD. This is the first disease to be treated using pluripotent stem cell-derived RPE and, therefore, forms a fundamental foundation and advancements in regenerative medicine involving cell therapy for the treatment, repair, or regeneration of any dysfunctional cell or tissue. Ever since the discovery of ESCs in 1981^108^ and iPSCs in 2006,^109^ there has been a remarkable expansion in the use of ESC and iPSC for treating a wide range of diseases like AMD and other genetic or metabolic diseases.^110,111^ However, AMD is the first disease that has been extensively explored for treatment using iPSC or ESC owing to its unique niche, which prevents further deterioration of the condition and possibly helps in restoring vision to an extent.^112,113^ Additionally, the number of cells that have been used so far for human studies has hardly gone over 5 × 10^4^ to 2 × 10^5^, which means the requirement for cells is quite less for transplantation and treatment of AMD in patients.^14^ Although cases may vary depending on the advanced stages of the disease progression but so far in human clinical trials cells up to 2 × 10^5^ have been used. Thus, making the GMP grade development of such cells a reproducible and facile process provided other molecular and genomic aspects are optimized and standardized. So far, a handful of clinical trials have been completed for treating macular degenerative diseases using stem cells derived RPE (Table 2). This is interesting as stem cell-derived cell products and their application in treating macular degenerative disease could possibly be the first treatment module that could be used in clinics soon.

**Table 2.** Tabular information on clinical trials where pluripotent stem cells (ESC, iPSC) were used as a cell source and injected into the patients with clinically diagnosed eye ailments like AMD, Stargardt’s macular dystrophy, RD, and RP.

Clinical trial status	Name of the condition	Clinical trial number	Study type	Number of patients	Stem cell type	Cell type	Cell number	Phase	Delivery route	Follow-up period
Completed	Dry AMD	NCT01344993	Interventional	13	hESC	MA09-hRPE	Cohort 1—50 000 cells transplanted; cohort 2—100 000 cells transplanted; cohort 3—150 000 cells transplanted; cohort 4—200 000	Phase I/II open-label, non-randomized, sequential, multi-center clinical trial	Sub retinal	12 Months
Completed	Stargardt macular degeneration	NCT01345006	Interventional	13	hESC	MA09-hRPE	Cohort 1—50 000 cells transplanted; cohort 2—100 000 cells transplanted; cohort 3—150 000 cells transplanted; cohort 4—200 000	Phase I/II open-label, non-randomized, sequential, multi-center clinical trial	Sub retinal	12 Months
Completed	AMD Stargardt macular degeneration	NCT02903576	Interventional	6	hESC	RPE	100 000	Phase I/II open-label, non-randomized,	Sub-retinal	12 Months
Completed	AMD Stargardt macular degeneration	NCT02903576	Interventional	15	hESC	RPE-Patch	100 000	Phase I/II open-label, non randomized,	Sub-retinal	12 Months
Completed	Stargardt’s macular dystrophy	NCT02941991	Observational	12	hESC	RPE	Cohort 1—50 000 cells transplanted; cohort 2—100 000 cells transplanted; cohort 3—150 000 cells transplanted; cohort 4—200 000	Phase I/II, open-label, multi-center, prospective	Sub-retinal	48 Months

The first study of ESC-derived RPE transplantation in humans for the testing of the safety in two patients with Stargardt’s macular dystrophy and dry AMD was published in 2012.^56^ In this study, the authors used hESC line MA09 for derivation of RPE. RPE derivation strategy was the convectional methods of isolating spontaneously formed pigmented RPE during embryoid body culture in a feeder-based system. The RPE during expansion and culture was characterized for its karyotypic, phagocytosis assay testing, differentiation and purity was further confirmed by quantitative RT PCR and immunophenotypic for RPE and ESCs marker. A total of 50 000 RPE cells were transplanted subretinally and the patients were monitored for 4 months. Patients did not show any sign of RPE cell hyperproliferation, tumorigenicity, ectopic tissue formation, or rejection after 4 months follow-up period. Thus, confirming the short-term safety of transplantation of ESC-derived RPE in humans.^56^

Another study examined the mid-term to long-term safety of three dose cohorts (50 000, 1000 000, and 150 000) of ESC-derived RPE was transplanted subretinally in 9 dry AMD and 9 Stargardt’s macular dystrophy patients and followed up for 22 months. The preparation of ESC-derived RPE was based on the spontaneous development of RPE during embryoid culture followed by isolation purification and expansion. The study showed that after 22 months, the transplanted cells confirmed the safety and graft survival. In total, 72% of the patients showed increased pigmentation at the site of transplantation consistent with transplanted RPE. Best-corrected visual acuity was improved in 10 patients, improved or remained the same in 7 patients, and deteriorated in 1 patient. Overall, the transplanted RPE was correlated with the vison-related quality of life like general and peripheral vision, near, and distant activities increased in both atrophic AMD and Stargardt’s macular dystrophy.^58^

Delivery of RPE has been shown to have certain disadvantages like inefficient cell engraftment, and loss of functionality of transplanted RPE.^114^ To overcome this disadvantage, researchers have focused on the development of an RPE patch fabricated using a biocompatible polymer supporting the RPE. Transplantation of such RPE patch is more localized and overcomes the limitation of cell-based delivery as mentioned earlier. In this regard, first patch-based ESC-derived RPE delivery was performed in 2018 when the authors fabricated ESC-derived RPE on human-vitronectin-coated polyester membrane. The RPE patch was transplanted successfully in 1 eye of 2 patients with severe exudative AMD. The transplanted RPE patch showed survival integration and improvement in visual acuity in both patients over 12 months.^16^ Thus, presenting the first report on using a patch-based RPE delivery format for improving AMD-related complications.

iPSCs are another source of pluripotent cells that have the advantage of being autologous, genetically identical to the host, and immune match compared to ESC. The first report about the use of iPSC-derived RPE in human studies was given in 2017 when the authors transplanted iPSC-derived RPE sheets in a 77-year-old female patient of a subtype of neovascular AMD.^75^ A 1-year follow-up showed that there was no improvement or reduction in visual acuity without any severe adverse reaction. In 2017, the same group started another study to examine the delivery of allogenic iPSC-derived RPE in AMD patients injected subretinally.^115^ Currently, many clinical trials focusing on iPSC-derived RPE are undergoing and the increase in iPSC-derived RPE-based clinical trials over ESC underscores the utility of iPSC over ESC in achieving a long-term solution for cell therapy in AMD.

## Conclusions

Over the last 3-decade, advancement in understanding the developmental process of the eye and in particular RPE using advanced modern technology has provided critical information regarding the role of paracrine factors and signaling processes involved in the development, growth, and differentiation of RPE during the course of eye development in utero. Utilizing such information has helped in the development of procedures for the engineering of stem cell derived-RPE in vitro for therapeutic and disease modeling purposes. The advancement in the delivery of RPE has further the clinical applications for treating ophthalmological diseases like AMD. The role of biomaterials in the delivery of RPE is in a nascent stage and must be explored to modulate RPE function in vitro and couple its functional and physical behavior in vivo upon transplantation. Pluripotent stem cells-derived RPE appears to be the first regenerative therapy mode to be extensively explored for treating human disease in clinical trials. Therefore, it becomes essential to study not only the long-term safety and efficiency but also the fate of such RPE in vivo to predict the behavior and functionality and the effect of the immune system and nearby tissue on the transplanted cells.

The long-term effect of transplantation and the ability of cells to undergo de-differentiation warrants a detailed understanding of stem cells-derived RPE both in vitro and in vivo to harness the regenerative potential of cell therapy. The future scope of the stem cell derived RPE also requires modelling RPE development in vitro to precisely achieve mature RPE which in utero requires retinal tissue interactions.

## Data Availability

The data underlying this article will be shared on reasonable request to the corresponding author.
